# Supplementary material for: The genome sequence of a cuckoo wasp, Hedychridium roseum (Rossi, 1790)
Source: Wellcome Open Res. 2024 Aug 14;9:484. [Version 1] doi: 10.12688/wellcomeopenres.22881.1 (PMC11464970; doi:10.12688/wellcomeopenres.22881.1)
Supplement: Peer review history

# Reviewer report: Reviewer response for version 1

**DOI:** 10.21956/wellcomeopenres.25196.r101217

**Published:** 2024-10-09

**Authors:** Thomas W. R Harrop

**Affiliations:** 1 Melbourne Bioinformatics, The University of Melbourne, Melbourne, Victoria, Australia

This is an genome note from the DToL project for an assembly for the cuckoo wasp, *Hedychridium roseum. *The assembly was generated from a single female using long-read sequencing, scaffolded using Hi-C data and then manually curated. The metrics are consistent with a good assembly, the methods are complete, and I didn't notice any issues with the submission.

Are sufficient details of methods and materials provided to allow replication by others?

Yes

Is the rationale for creating the dataset(s) clearly described?

Yes

Are the datasets clearly presented in a useable and accessible format?

Yes

Are the protocols appropriate and is the work technically sound?

Yes

Reviewer Expertise:

Genomics

I confirm that I have read this submission and believe that I have an appropriate level of expertise to confirm that it is of an acceptable scientific standard.

# Reviewer report: Reviewer response for version 1

**DOI:** 10.21956/wellcomeopenres.25196.r99699

**Published:** 2024-09-30

**Authors:** Hollie Marshall

**Affiliations:** 1 University of Leicester, Leicester, UK

This article is a standard genome note from the Darwin Tree of Life, describing the new genome assembly of *Hedychridium roseum*.

 The description of the methods is excellent, allowing full reproduction of the pipeline and the results are clearly reported with all data publicly available. I have no comments for improvement.

Are sufficient details of methods and materials provided to allow replication by others?

Yes

Is the rationale for creating the dataset(s) clearly described?

Yes

Are the datasets clearly presented in a useable and accessible format?

Yes

Are the protocols appropriate and is the work technically sound?

Yes

Reviewer Expertise:

Genetics / epigenetics of non-model insect species.

I confirm that I have read this submission and believe that I have an appropriate level of expertise to confirm that it is of an acceptable scientific standard.
